# Activation of retinal glial (Müller) cells by extracellular ATP induces pronounced increases in extracellular H+ flux

**DOI:** 10.1371/journal.pone.0190893

**Published:** 2018-02-21

**Authors:** Boriana K. Tchernookova, Chad Heer, Marin Young, David Swygart, Ryan Kaufman, Michael Gongwer, Lexi Shepherd, Hannah Caringal, Jason Jacoby, Matthew A. Kreitzer, Robert Paul Malchow

**Affiliations:** 1 Department of Biological Sciences, University of Illinois at Chicago, Chicago, Illinois, United States of America; 2 Department of Biology, Indiana Wesleyan University, Marion, Indiana, United States of America; 3 Department of Ophthalmology & Visual Sciences, University of Illinois at Chicago, Chicago, Illinois, United States of America; University of Florida, UNITED STATES

## Abstract

Small alterations in extracellular acidity are potentially important modulators of neuronal signaling within the vertebrate retina. Here we report a novel extracellular acidification mechanism mediated by glial cells in the retina. Using self-referencing H+-selective microelectrodes to measure extracellular H+ fluxes, we show that activation of retinal Müller (glial) cells of the tiger salamander by micromolar concentrations of extracellular ATP induces a pronounced extracellular H+ flux independent of bicarbonate transport. ADP, UTP and the non-hydrolyzable analog ATPγs at micromolar concentrations were also potent stimulators of extracellular H+ fluxes, but adenosine was not. The extracellular H+ fluxes induced by ATP were mimicked by the P2Y1 agonist MRS 2365 and were significantly reduced by the P2 receptor blockers suramin and PPADS, suggesting activation of P2Y receptors. Bath-applied ATP induced an intracellular rise in calcium in Müller cells; both the calcium rise and the extracellular H+ fluxes were significantly attenuated when calcium re-loading into the endoplasmic reticulum was inhibited by thapsigargin and when the PLC-IP3 signaling pathway was disrupted with 2-APB and U73122. The anion transport inhibitor DIDS also markedly reduced the ATP-induced increase in H+ flux while SITS had no effect. ATP-induced H+ fluxes were also observed from Müller cells isolated from human, rat, monkey, skate and lamprey retinae, suggesting a highly evolutionarily conserved mechanism of potential general importance. Extracellular ATP also induced significant increases in extracellular H+ flux at the level of both the outer and inner plexiform layers in retinal slices of tiger salamander which was significantly reduced by suramin and PPADS. We suggest that the novel H+ flux mediated by ATP-activation of Müller cells and of other glia as well may be a key mechanism modulating neuronal signaling in the vertebrate retina and throughout the brain.

## Introduction

Modulation of synaptic transmission and regulation of cellular excitability play essential roles in nervous system function. Recently, much interest has focused on the hypothesis that glial cells, support cells in the brain, may normally play a key role in modulating such neuronal activity. Activation of glial cells is well known to induce increases in calcium in the cytosol of these cells, and a variety of “gliotransmitters” have been suggested to be released by glial cells upon such activation. Among the agents typically considered as gliotransmitters are glutamate, GABA, D-serine, and ATP. As noted in a number of reviews, the mechanisms that govern the release of these gliotransmitters and their precise roles in regulating neuronal activity remain highly controversial ([1–4]).

An especially potent and underappreciated mechanism of modulation of synaptic transmission and cellular excitability involves small alterations of extracellular levels of H+. A large body of evidence suggests that changes in extracellular H+ levels in the outer retina may play an important role in shaping the response properties of retinal neurons (cf. [5,6] for review). Enriching the pH buffering capacity of the extracellular solution blocks the ability of horizontal cells to induce shifts in the calcium currents in photoreceptors and reduces calcium signals in photoreceptor synaptic terminals [7,8]. External alkalinization increases photoreceptor calcium currents and shifts the activation of the calcium conductance to more negative voltages [7,9]. Simultaneous paired recordings from horizontal and photoreceptor cells in retinal slices reveal that direct depolarization of horizontal cells induces a rightward shift of the calcium conductance activation curve that is abolished by enhancing the extracellular pH buffering capacity [10]. Particularly strong evidence for a role for H+ in providing inhibition onto photoreceptor synaptic terminals has come from experiments fusing the H+-sensitive fluorescent molecule pHluorin onto the extracellular portion of cone calcium channel subunits expressed in photoreceptors. Measurements in the intact retina of such transgenic zebrafish reveal alterations in fluorescence whose magnitude, direction and spatial dependence are consistent with the hypothesis that changes in H+ significantly impact retinal signals [11].

Here we show that Müller cells, the radial glia of the retina, are potent sources of extracellular acidification when activated by low concentrations of extracellular ATP. These cells extensively enwrap and envelop all retinal neurons and their synaptic interconnections, and are well placed to be able to modulate the release of neurotransmitter by retinal neurons. Using self-referencing H+-selective microelectrodes, we find that micromolar levels of extracellular ATP promote a significant increase in extracellular H+-flux not dependent upon bicarbonate. This novel ATP-induced extracellular H+-flux can be detected at the outer synaptic layer, where photoreceptors and second-order neurons make synaptic connections, as well as at the inner synaptic layer, where bipolar and amacrine cells pass their signals to ganglion cells, the output neurons of the eye. Moreover, the ATP-induced increase in extracellular H+ flux is highly conserved across a wide number of evolutionarily distant species and includes Müller cells isolated from human retinae. Our results suggest that this glial-cell mediated extracellular acidification may be a key regulator of neurotransmission in the retina and throughout the nervous system.

## Materials and methods

### Isolated cells

All experiments/procedures were conducted following animal care protocols approved by the Institutional Animal Care and Use Committee at Indiana Wesleyan University and the University of Illinois at Chicago. Larval tiger salamanders (*Ambystoma tigrinum*) 5–10 inches were obtained from Charles D. Sullivan Co. Inc. (Nashville, TN) and housed at 4–7°C for up to 2 months. Lampreys and tissues of Sprague-Dawley rats were provided by Dr. Simon Alford’s laboratory, University of Illinois at Chicago. Channel catfish (*Ictalurus puntatus*), 6–10 inches in length, were obtained from Osage Catfisheries, MO. Skates were obtained from the Marine Biological Laboratory in Woods Hole, Massachusetts. Macaque eyes (*Macaca fascicularis* and *Macaca mulatta*) were provided by the Biologic Resource Laboratories (BRL) at the University of Illinois at Chicago. Post-mortem human donor eyes were provided by Eversight Illinois.

Tiger salamanders were anesthetized for 20 min (3 g/gal MS-222 (Argent), 7.5 g/gal NaHCO3) and double-pithed. Eyes were removed and hemisected; eyecups were immersed in 5 ml dissociation solution (mM: 110 NaCl, 1.5 KCl, 0.5 MgCl_2_, 10 HEPES, 10 glucose, pH 7.40) containing 1.2 mg/ml papain (LS003119, Worthington) and 0.8 mg/ml cysteine. The retina was peeled off and let sit in the papain solution on a gently vibrating plate for 25 min, then rinsed 6–8 times in dissociation solution and mechanically triturated with a 5-ml pipette. One drop of the cell suspension was placed in 35-mm culture dishes (Falcon 3001) preloaded with plating saline containing (mM): 110 NaCl, 2.5 KCl, 2.0 CaCl_2_, 2.0 MgCl_2_, 10 glucose, 10 HEPES, and then stored at 10°C during the day of experiments. Prior to conducting experiments, the solution was replaced with 3 ml of Ringer’s solution of identical composition except that the concentration of HEPES was reduced to 1 mM with the difference in osmolarity compensated by the addition of sucrose. pH was adjusted to 7.60, typical of salamander Ringer’s solutions. This concentration of HEPES was chosen to provide sufficient buffering power to maintain a reasonably stable extracellular pH in the dish while maximizing the likelihood of observing changes in extracellular H+ adjacent to cells. No bicarbonate or CO2 were added to the media.

A similar protocol was followed for dissociations of lamprey, rat, catfish, and skate using appropriate Ringer’s solutions. In dissociations of human and monkey retinal cells, enzymatic digestion with papain was done with the retina maintained at 37°C and gently bubbling the Ringer’s solution with oxygen. During recordings, the solution was replaced with one containing 1 mM HEPES; no oxygen, CO2 or bicarbonate were added during experiments.

### Retinal slice preparation

Animals were euthanized as described above. The eyecup was hemisected and the posterior eyecup with the retina attached cut in half with a razor blade. The eyecup was then flipped onto Millipore filter paper and the sclera removed. The retina and filter paper were placed on a Millipore filter holder and suction applied to flatten the retina while dropping on Ringer’s solution to keep the tissue moist. The filter paper was then trimmed and placed on a custom-made slicing chamber filled with saline with the paper secured at either end with vacuum grease [12]. The tissue was sliced using a Stoelting slicer yielding 200–250 μm sections. Retinal slices were transferred to a recording chamber by creating a fluid bridge between the slicing and recording chambers and turned 90 degrees.

### Self-referencing measurements of extracellular H+ fluxes

H+-selective microelectrodes were prepared as previously described and used in a self-referencing format to record differential voltage measurements reflective of extracellular H+ flux [13–20]. To make a self-referencing measurement from isolated cells, the tip of an H+-selective electrode was placed 1–2 μm from the cell membrane and the voltage of the H+-selective electrode recorded; the microelectrode was then moved 30 μm away parallel to the bottom of the dish and a second reading taken; the second reading was subtracted from that obtained close to the cell. The electrode was moved alternately between these two points at a rate of 3 Hz to minimize stirring of the solution. This differential recording procedure eliminates slow electrical drift present in ion selective electrodes, increases their useful sensitivity by ~1000X [14,21], and allows for the detection of an extracellular H+ flux, the magnitude of which depends in part on the distance the sensor travels in its movement to and from the cell. The differential measurements made in this study are presented as the difference in the voltage reported at the two locations, i.e. “Δ μV”, from which fluxes can be calculated as described in detail in Smith et al. [13] and Molina et al. [15]. For recordings from retinal slices, the microelectrode was placed ~ 2 μm above the outer plexiform layer and alternately moved to a position 30 μm vertically above the slice. Control background differential readings were made in every experiment with microelectrodes far removed from the cell (200 μm) or tissue (600 μm); recordings in which differential responses were not close to zero at these control locations were discarded.

To prepare H+-selective microelectrodes, borosilicate capillaries were pulled to tip diameters of 2–4 μm, silanized, and backfilled with a solution containing 100 ml KCl and 10 mM HEPES adjusted to pH 7.40 with KOH. The pipette tip was then placed in contact with a highly selective H+ resin (hydrogen ionophore 1, cocktail-B, Fluka Co.), and ~ 30 μm of resin was drawn into the tip by gentle suction applied to the back of the pipette. The pH resin employed here is highly selective for H+ (~ 10^9^ times more sensitive to H+ than to Na^+^ or K^+^; Fluka Co.). Extracellular voltage fields generated by isolated cells are unlikely to contribute to signals, as such fields are usually in the nanovolt range and below the sensitivity of ion-selective self-referencing probes [13,22]. Potentials from local boundary conditions associated with membrane surface charges [23,24] are also unlikely to be the source of the signals. These fields typically drop with the Debye length and do not extend into the medium by more than tens of angstroms (cf [25]); our H+-selective microelectrodes were located at least 1 μm away from the surface of isolated cells. We also conducted control experiments using pipettes filled with the same hydrophobic base (NPOE containing 1% potassium-tetrakis) but lacking the H+-selective ionophore. In recordings from 5 cells, addition of 10 μM ATP (adenosine 5’-triphosphate) failed to induce a significant increase in acidity (38 ± 13 μV compared to 30 ± 8 μV, respectively; p = 0.63). This slight and insignificant voltage increase was only about 7% of the voltage increase induced by 10 μM ATP and detected with normal H+-selective micropipettes. These control experiments indicate that the ATP-induced changes detected with H+-selective electrodes reflect changes in extracellular H+ and not alterations in extracellular voltages.

We also conducted control experiments in slice preparations with pipettes filled with the same hydrophobic base (NPOE containing 1% potassium-tetrakis) but lacking the H+ selective ionophore. The mean signal elicited by 100 μM ATP with such pipettes was only 1.5–3% (N = 6) of the voltage change observed from slices recordings with control pipettes filled with the H+ ionophore (N = 8). These results suggest that in slice preparations, the contribution from extracellular voltages was also quite small.

Recordings were made in mechanically quiet solutions since superfusion washes away the H+ gradient between the two points of electrode movement. Drugs were applied by hand pipettor to either dish or slice; in all such experiments, a control bolus of Ringer’s solution without drug was first applied to ensure that responses observed did not result from simple mechanical application and stirring of the solution. For some experiments with cells and slices, drugs were applied or washed out by a full solution exchange: the majority of the solution in the culture dish was drawn up through tubing attached to a syringe, leaving behind just enough solution coating the dish’s bottom to ensure a continuous aqueous environment for the cells; full solution exchanges were ordinarily repeated three times. Recordings were performed in plating solution in which 10 mM HEPES was substituted with 1 mM HEPES with the difference in osmolarity compensated by the addition of sucrose. All experiments were conducted at room temperature (~18–25°C).

Control experiments were conducted to ensure that drugs at the concentrations applied did not alter the sensitivity of the H+ sensors; microelectrodes were calibrated with standard pH solutions of 6.00, 7.00 and 8.00 and the Nernstian slope of electrodes determined. None of the agents used in this study altered the slope of the electrodes at the concentrations used. We note however, that concentrations of 4, 4’-Diisothiocyanatostilbene-2,2-disulfonic acid (DIDS) of 500 μM or greater did reduce the sensitivity of the sensors, while concentrations of 300 μM or less had no significant effect (slopes per pH unit in μV: Ringer’s solution alone, 57 ± 0.5 (N = 21); DIDS at 100 μM, 60 ± 0.8 (N = 11), 250 μM, 56 ± 1.7 (N = 5); 300 μM, 58.6 ± 1 (N = 7); 500 μM, 42 ± 0.9 (N = 9); 1 mM, 28 ± 1 (N = 5).

### Calcium imaging

The calcium indicator dye Oregon Green 488 BAPTA-1, AM (Invitrogen Molecular Probes, Eugene, OR, USA), was employed to monitor intracellular calcium changes in isolated cells. A stock solution was created by dissolving 50 μg Oregon Green in 20 μl dimethylsulfoxide (DMSO) with 20% pluronic F-127 detergent (ThermoFisher, P-3000MP). Oregon Green stock solutions were diluted in salamander Ringer’s solution to a final concentration of 5 μM. Cells were incubated in Oregon Green-containing Ringer’s solution in the dark for 10–14 min at room temperature. Cells were imaged following wash-off with dye-free Ringer’s solution. Epifluorescent images were collected from a CCD camera (Zeiss 503 mono) mounted onto a compound microscope (Zeiss Axio Observer Z1). A Colibri LED system stimulated loaded cells with 470 and 505 nm light. Images were filtered through a 535 nm bandpass filter (30 nm, 46 HE YFP) with binning 2 x 2. Images were acquired every 4 s with ~150 ms exposure using Axiovision software.

All chemicals were obtained from Sigma Aldrich except the following: 4, 4’-Diisothiocyanatostilbene-2,2-disulfonic acid disodium salt, DIDS (6588, Setareh Biotech, LLC); Pyridoxal phosphate-6-azo(benzene-2,4-disulphonic acid), PPADS (ab120009), Suramin (B2070061), U-73343 (B5110027), and 2-Aminoethoxydiphenylborane, 2-APB (B5060114) were purchased from ABCAM. DL-threo-β-Benzyloxyaspartic acid, DL-TBOA (1223) was purchased from Tocris.

### Data treatment and statistical analysis

Data are presented as raw microvolt values rather than converting to a value for pH, since the data obtained reflect proton fluxes—differences between proton signals obtained over a known distance of 30 μm—not concentrations. Moreover, since protons extruded from isolated cells diffuse into the vast ocean of extracellular solution, a calculation of estimated values for changes in pH flux gives an inappropriately small estimate for the changes that would be expected to be observed in the small confines of the extracellular spaces in normal tissue. Thus, the signals reported here likely represent significant underestimates of the actual changes in proton concentration that would result in intact tissue.

Student’s paired t-tests were used to determine statistical significance in all experiments except where noted. Data are presented throughout as mean ± SEM with individual P values provided. For most experiments, standing flux values reflect the average of the full length of the recording prior to drug application. Transient electrical artifacts resulting from opening the Faraday cage and dropping in drugs by hand pipette have been eliminated from data traces and appear as spaces in the traces. In some cases, the ATP-induced acidification took some time to reach a plateau; in these instances, the average of the plateau during the last 20 points was taken. All data were statistically analyzed and graphically displayed using Prism 5 (GraphPad Software). In histograms, a comparison with a P value of 0.01 or less is indicated by two asterisks.

## Results

**Fig 1:**
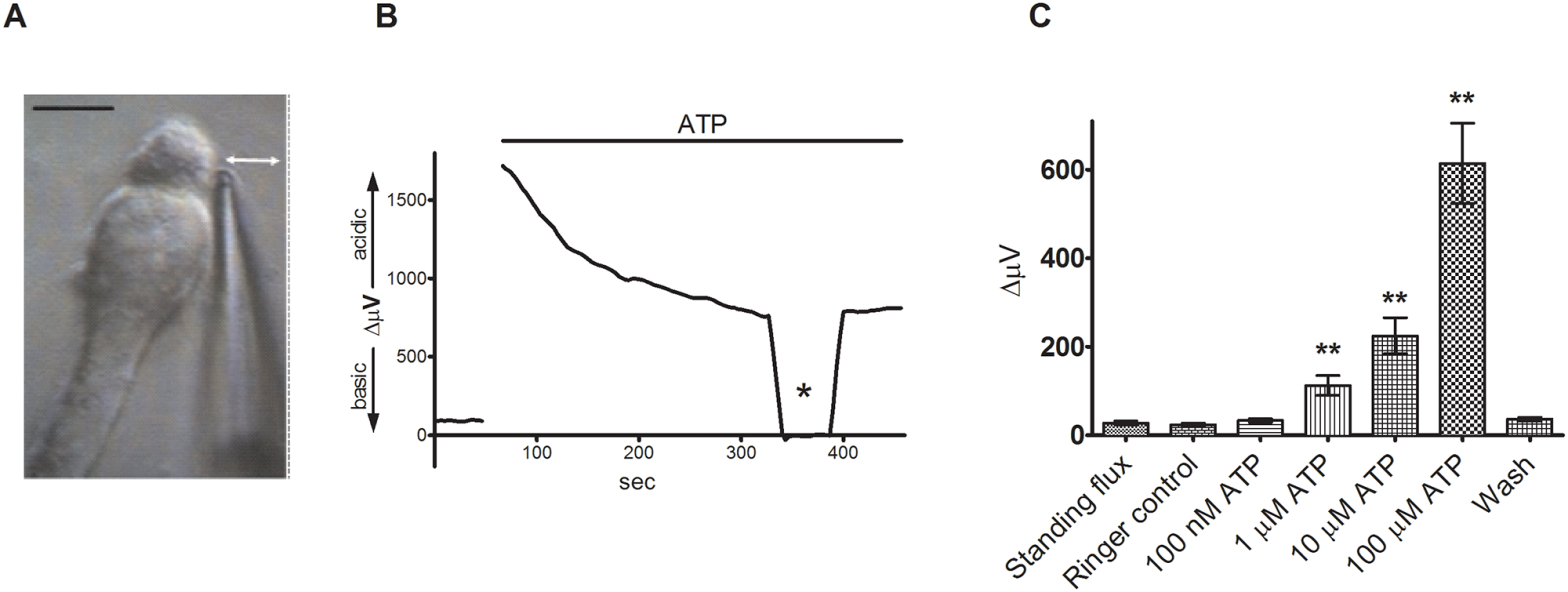
Extracellular ATP induces a significant increase in extracellular H+ flux from isolated Müller cells. (A) An isolated Müller cell with a H+-selective microelectrode positioned next to the apical end of the cell. Scale bar: 20 μm; double-headed arrow depicts the direction of electrode movement as it alternately records the potential established by protons adjacent to the cell and 30 μm away. (B) Response from a single isolated Müller cell to 100 μM ATP. Top bar represents duration of drug application. Asterisk: background control readings taken 200 μm above the cell. (C) Mean data from 8 trials in response to various extracellular ATP concentrations: error bars represent SEMs; data was analyzed with a paired 2-way t-test. Bars represent mean values ± SEM.

Isolated tiger salamander Müller cells were identified by their distinctive morphology, characterized by an apical end with a tuft-like outer process, a cell body, an elongated and relatively thick inner process, and a wide stubby end foot [26]. H+-selective microelectrodes were typically placed approximately 1–2 μm from the cell membrane where the apical tip of the cell and cell body fused (Fig 1A); a differential voltage reflecting H+ flux was obtained by moving the electrode to a position 30 μm distant and subtracting the recordings obtained at the near and distal positions. Recordings were obtained in a Ringer’s solution containing 1 mM HEPES as the primary extracellular pH buffer; no bicarbonate was added to the bath and no bubbling with CO2 was involved. Unstimulated cells typically displayed a standing differential signal averaging 28 ± 4 μV (N = 8), indicating that the area adjacent to the cell membrane was slightly more acidic than the point 30 μm away. The trace in Fig 1B shows that 100 μM ATP induced a marked increase in extracellular H+ flux adjacent to the cell membrane. The mean differential electrode response from salamander Müller cells was 112 ± 22 μV to 1 μM ATP, p = 0.004; 225 ±41 μV to 10 μM ATP, p = 0.002, and 614 ± 91 μV to 100 μM ATP, p = 0.0003 (N = 8).

The non-hydrolyzable ATP analogue ATPγs was also potent in inducing an increase in extracellular H+ flux. In recordings from nine cells, 100 μM ATPγs increased the signal associated with H+ flux from a baseline reading of 57 ± 10 μV to 239 ± 31 μV (p = 0.0002). ADP (adenosine 5’-diphosphate) and UTP (uridine 5’-triphosphate) also elicited large increases in extracellular H+ flux from Müller cells. 100 μM UTP induced a differential response of 762 ± 256 μV from a mean standing flux reading of 61 ± 23 μV (p = 0.003, N = 4); 10 μM UTP acidified the extracellular space from a standing flux of 148 ± 16 μV to 264 ± 27 μV (p = 0.002, N = 6). In six other tested cells, 100 μM ADP caused an increase in H+ flux, with the signal increasing from a standing flux of 41 ± 6 μV to 235 ± 18 μV (p<0.0001, N = 6); 10 μM ADP increased the signal from from 106 ± 32 μV to 178 ± 21 μV (p = 0.03, N = 4). However, 100 μM adenosine did not alter extracellular proton fluxes. In 5 cells examined, the signal obtained in the presence of 100 μM adenosine (8 ± 3 μV) was not significantly different from the standing baseline signal of 7 ± 3 μV (p = 0.77); in these same cells, 10 μM ATP increased the signal to 115 ± 20 μV (p = 0.006).

**Fig 2:**
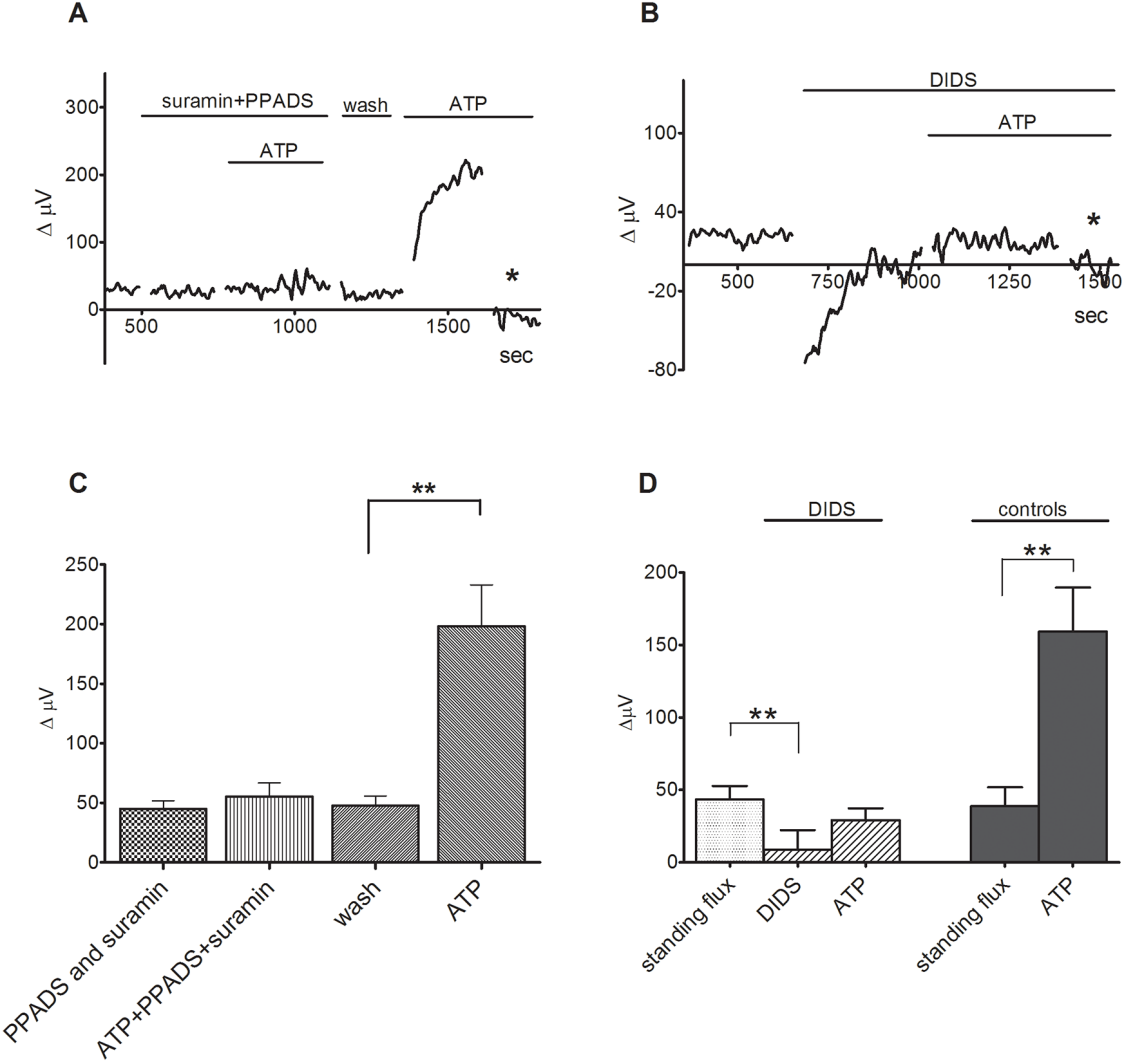
Inhibition by suramin, PPADS and DIDS significantly reduces the ATP-induced increase in extracellular H+ flux from isolated Müller cells. (A) A representative trace from a single Müller cell shows a significant increase in H+ flux in response to 10 μM ATP that is significantly reduced by the ATP receptor blockers 200 μM PPADS and 200 μM suramin; asterisk indicates a background control reading. (B) 300 μM DIDS, which inhibits anion transport, significantly reduces the increase in H+ flux in response to 10 μM ATP. (C) Mean responses to 10 μM ATP with or without suramin and PPADS in the bath; N = 7, error bars represent SEMs. (D) Mean responses to 10 μM ATP in the presence of 300 μM DIDS (N = 6) and in the absence of DIDS, N = 5 (controls).

The purinergic receptor antagonists suramin and PPADS significantly reduced the increase in extracellular H+ flux induced by extracellular ATP. Fig 2A shows responses from one Müller cell to application of 10 μM ATP first in the presence of 200 μM suramin and 200 μM PPADS and then after washout of the blockers; the cell produced little response when the blockers were present, but ATP produced a robust increase in extracellular H+ flux following washout of the blockers. The mean standing signal from seven cells in the presence of the blockers was 45 ± 7 μV before and 55 ± 8 μV following the application of 10 μM ATP (p = 0.13); upon washout of suramin and PPADS, the baseline signal was 48 ± 8 μV and 10 μM ATP now elicited a response of 198 ± 35 μV (p = 0.002; Fig 2C). The lack of an effect by adenosine, coupled with the reduction of the ATP-induced increase in H+ flux by PPADS and suramin and activation by UTP and ADP, suggest that the changes in H+ flux are mediated by activation of metabotropic P2Y receptors known to be present on these cells [27]. We also examined the effect of the compound MRS 2365, reported to be a selective agonist of P2Y1 receptors ([28,29]), on H+ flux from isolated Müller cells. In seven cells tested, 1 μM MRS 2365 increased the signal from H+-selective self-referencing sensors from a pre stimulus level of 53 ± 6 μV to 253 ± 65 μV (p = 0.012); in the same cells, 10 μM ATP applied after wash out of MRS 2365 increased the signal associated with H+ flux from 99 ± 6 μV to 453 ± 6 μV (p = 0.005). In a separate population of eight cells, we found that the effects of MRS 2365 could be blocked by the P2Y1 antagonist MRS 2179 [29]. In this population, 1 μM MRS 2365 significantly increased the signal from a baseline level of 75 ± 17 μV to 189 ± 37 μV (p = 0.009). Washout of MRS 2365 led to a reduction of the signal to 96 ± 17 μV, and reapplication of the MRS 2365 in the presence of 100 μM of the P2Y1 antagonist MRS 2179 now failed to produce a significant increase in the signal (113 ± 29 μV; p = 0.37).

The ATP-induced increase in H+ flux was also inhibited by DIDS, a non-selective anion exchange antagonist. 300 μM DIDS abolished the ability of 10 μM ATP to alter extracellular H+ levels (Fig 2B and 2D). The mean standing signal in the presence of DIDS was 9 ± 14 μV before and 29 ± 8 μV after the application of 10 μM ATP (p = 0.06, N = 6). In the absence of DIDS, bath application of 10 μM ATP significantly increased the signal from self-referencing electrodes from 39 ± 13 μV to 159 ± 30 μV (p = 0.004, N = 5). DIDS also significantly reduced the increase in H+ flux normally induced by 100 μM ATP. The standing H+ flux was also often reduced after exposure of the cells to DIDS. On average, the standing signal decreased from 43 ± 9 μV before to 9 ± 14 μV after exposure of the cells to DIDS (p = 0.028). Interestingly, the increase in the signal induced by extracellular ATP was not significantly reduced by 300 μM SITS, another agent known to block certain anion channels and transporters. In 8 cells bathed in 300 μM SITS, 10 μM ATP significantly increased the H+ flux signal from a baseline value of 26 ± 7 μV to 169 ± 26 μV (p = 0.0002). 300 μM SITS also failed to alter the standing H+ flux in these same cells; prior to application, the baseline reading was 12 ± 4 μV, and in the presence of 300 μM SITS, the flux was measured at 26 ± 7 μV (p = 0.10). In the same cells, 300 μM DIDS abolished the ability of 10 μM ATP to alter extracellular H+ flux. The signal of 21 ± 7 μV in the presence of 300 μM DIDS was statistically indistinguishable from that seen when 10 μM ATP was added in the presence of 300 μM DIDS (25 ± 2 μV; p = 0.55).

Importantly, the effect of DIDS is not likely to be due to its known ability to block the sodium-bicarbonate cotransporter present in Müller cells [30,31]. In the present experiments, all recordings were made in solutions containing 1 mM HEPES as the only significant pH buffer; no bicarbonate was added to the bath, nor was CO2 bubbled in the solution. Under such conditions, sodium-coupled transport of bicarbonate should not significantly contribute to acid/base fluxes. We confirmed the lack of contribution from voltage-dependent sodium-bicarbonate exchange by examining the effects of high extracellular potassium on H+ fluxes. Depolarization of Müller cells with high extracellular potassium is known to activate the voltage-dependent sodium bicarbonate cotransporter and produce extracellular acidifications when bicarbonate is present, but not when bicarbonate is absent [19,30,31]. In our control experiments, application of 50 mM KCl failed to alter extracellular H+ flux: in 5 cells tested, a signal of 10 ± 3 μV was observed upon application of Ringer’s solution, and a signal of 18 ± 6 μV detected after addition of 50 mM KCl (p = 0.18, N = 5).

**Fig 3:**
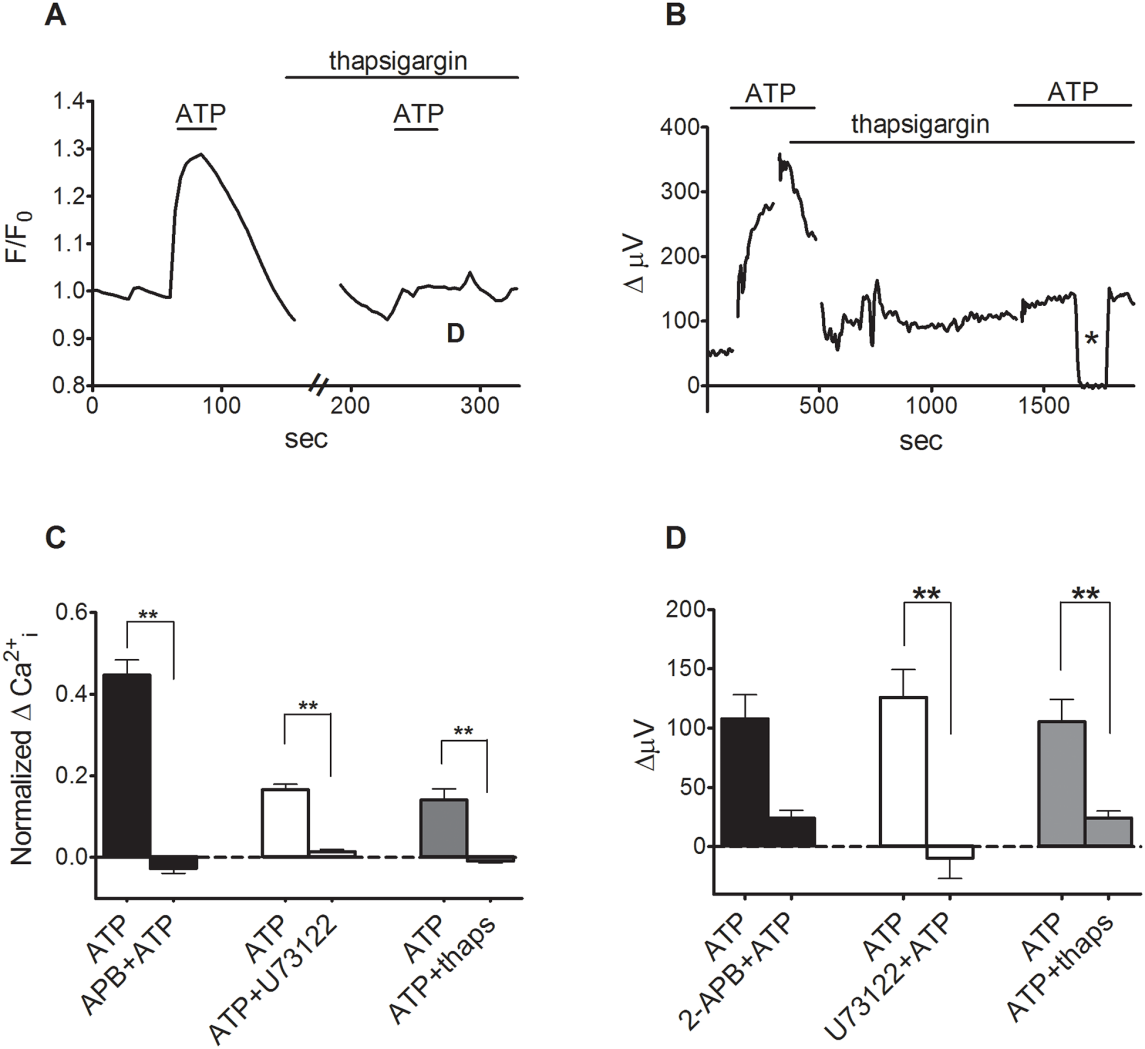
ATP-induced changes in intracellular calcium and extracellular H+ flux. (A) 1 μM ATP increased intracellular calcium as reported by changes in the fluorescence of the calcium indicator Oregon Green. Subsequent ATP-induced alterations in Oregon Green fluorescence were blocked by 1 μM thapsigargin applied for 15 minutes. (B) A representative single self-referencing recording from an isolated Müller cell demonstrating that thapsigargin also reduced the increase in H+ flux induced by 1 μM ATP from Müller cells; asterisk: background control. (C) Mean responses from imaging data for ATP induced responses in 2-APB, U73122 and thapsigargin. Data was normalized to the last fluorescence data point before treatment. (D) Mean responses with self-referencing. ATP readings represent absolute recorded values minus values of the standing flux.

The increase in extracellular H+ flux from Müller cells exposed to bath-applied ATP was dependent on rises of cytosolic calcium from intracellular stores. All agents tested here that led to an increase in extracellular H+ flux also induced an increase in intracellular calcium as measured by alterations in the fluorescence of the calcium indicator dye Oregon Green. In 18 cells tested, application of 100 μM ATPγs increased the fluorescence above background by a factor of 1.27 ± 0.02 (fluorescence divided by background fluorescence prior to stimulation; p <0.0001), while in the same cells following washout of the ATPγs, an application of 10 μM ATP increased fluorescence to 1.29 ± 02 over background (p < 0.0001). In a second population of 14 cells, 50 μM ADP increased Oregon Green fluorescence to 1.66 ± 0.02 over background (p < 0.0001); following washout of ADP, 10 μM ATP increased fluorescence to 1.50 ± 0.02 over background in the same cells (p < 0.0001). We also found that 1 μM of MRS 2365, the putative P2Y1 receptor agonist, also increased Oregon Green fluorescence over pre-stimulus levels in a separate population of 14 other cells by a factor of 1.29 ± 0.03 (p < 0.0001), while in the same cells following washout of the MRS compound, 10 μM ATP increased fluorescence by a factor of 1.33 ± 0.04 (p < 0.0001). In a separate population of 13 other cells, the increase in calcium-dependent fluorescence over background induced by 1 μM MRS 2365 (1.5 ± 0.04, p < 0.0001) was abolished when the same cells were challenged with 1 μM MRS 2365 in the presence of 100 μM of the P2Y1 receptor blocker MRS 2179 (1.0 ± 0.02, p = 0.75). Moreover, thapsigargin, which inhibits calcium ATPases on the endoplasmic reticulum and prevents reloading of calcium into the endoplasmic reticulum, effectively eliminated ATP-induced increases in intracellular calcium reported by Oregon Green (p = 0.0008, N = 10; Fig 3A and 3C). Thapsigargin also significantly reduced the ATP-induced increases in H+ flux measured with self-referencing electrodes (Fig 3B and 3D). In 6 trials the mean response to 1 μM ATP of 105 ± 19 μV was reduced to 24 ± 6 μV in the presence of 1 μM thapsigargin, (p = 0.005). In addition, the intracellular calcium rise (p <0.0001, N = 9) and increase in extracellular H+ flux (p = 0.026, N = 5) normally produced by ATP were also inhibited by 100 μM 2-APB, which blocks IP3 receptor-mediated calcium flux (Fig 3C and 3D). Finally, 25 μM of U-73122, which inhibits activation of G-protein-coupled PLC, also significantly reduced intracellular calcium signals (p<0.0001, N = 9) and extracellular changes in H+ flux (p = 0.004, N = 7).

**Fig 4:**
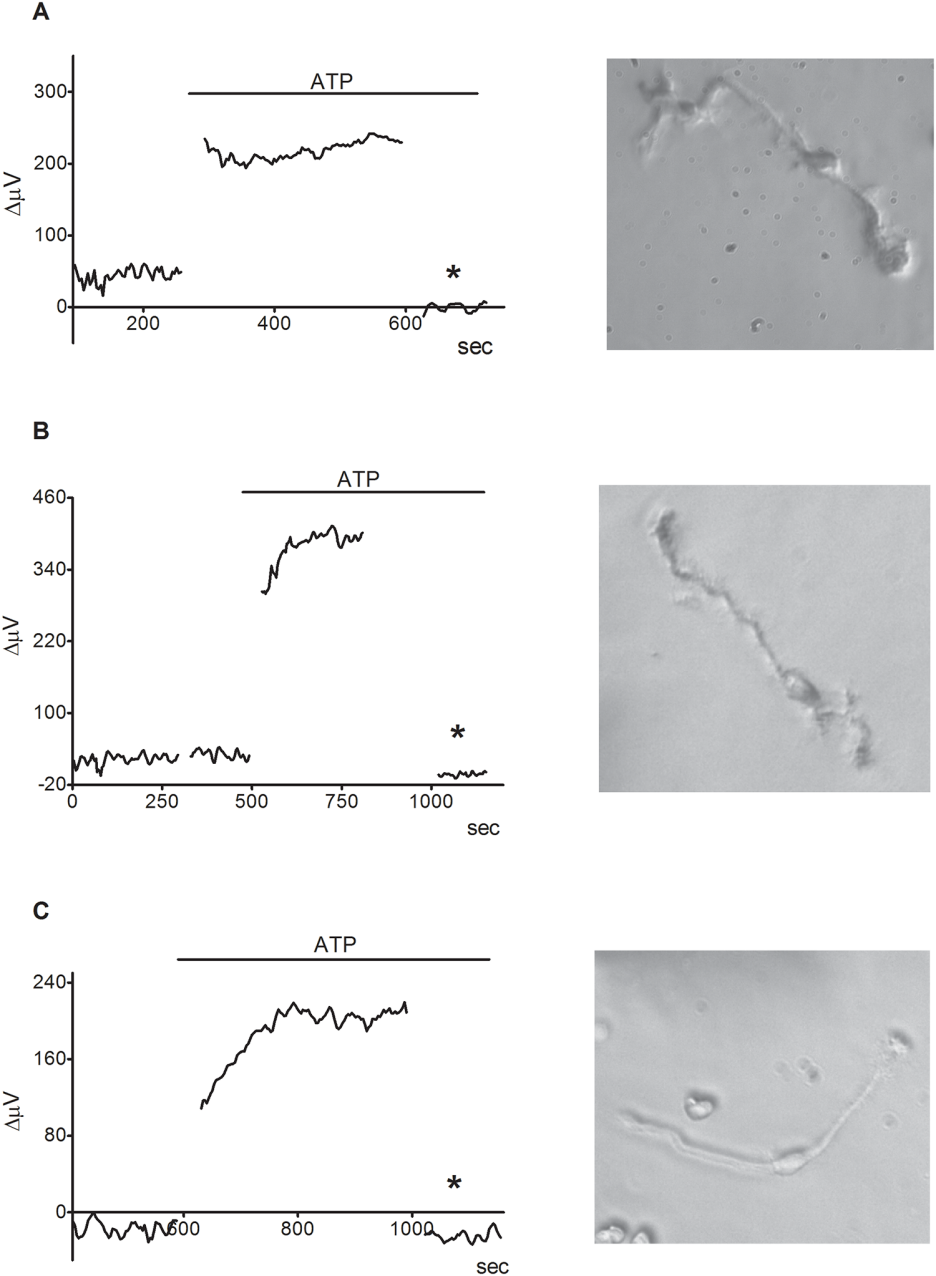
100 μM ATP causes an increase in extracellular H+ flux from isolated Müller cells from human retina (A), Sprague-Dawley rat retina (B) and lamprey retina (C). An image of an isolated Müller cell from each species is to the right of each respective trace.

The increase in extracellular H+ flux induced by bath-applied ATP was also observed in Müller cells isolated from a number of other species. Fig 4 shows responses from Müller cells isolated from human donor tissue, rat, and lamprey. Seven out of nine human Müller cells (~78%) showed clear responses to ATP with detectable increases in extracellular H+ flux (Fig 4A). The mean standing flux increased from 62 ± 9 μV before to 257 ± 41 μV after application of 100 μM ATP, (p = 0.001). Five out of six (~83%) rat Müller cells responded to 100 μM ATP (Fig 4B). The mean standing signal was 5 ± 7 μV, and 100 μM ATP induced an increase in the signal to 262 ± 54 μV, a difference that was also statistically significant (p = 0.006). Six out of seven lamprey Müller cells (~86%) also responded to 100 μM ATP (Fig 4C). The signal increased from -1 ± 13 μV in plain Ringer’s solution to 88 ± 21 μV in response to ATP (N = 7, p = 0.022).

Similar results were obtained from isolated Müller cells of skate, channel catfish, and two Macaque species, 5 cells from Macaca mullata (baseline signal 14 ± 8 μV; in ATP, 306 ± 83 μV; p = 0.024) and six cells from Macaca fascicularis (35 ± 6 μV; response of 3 cells to 85 μM ATP was 222 ± 48 μV and response of 3 cells to 100 μM ATP was 318 ± 83 μV, p = 0.004).

**Fig 5:**
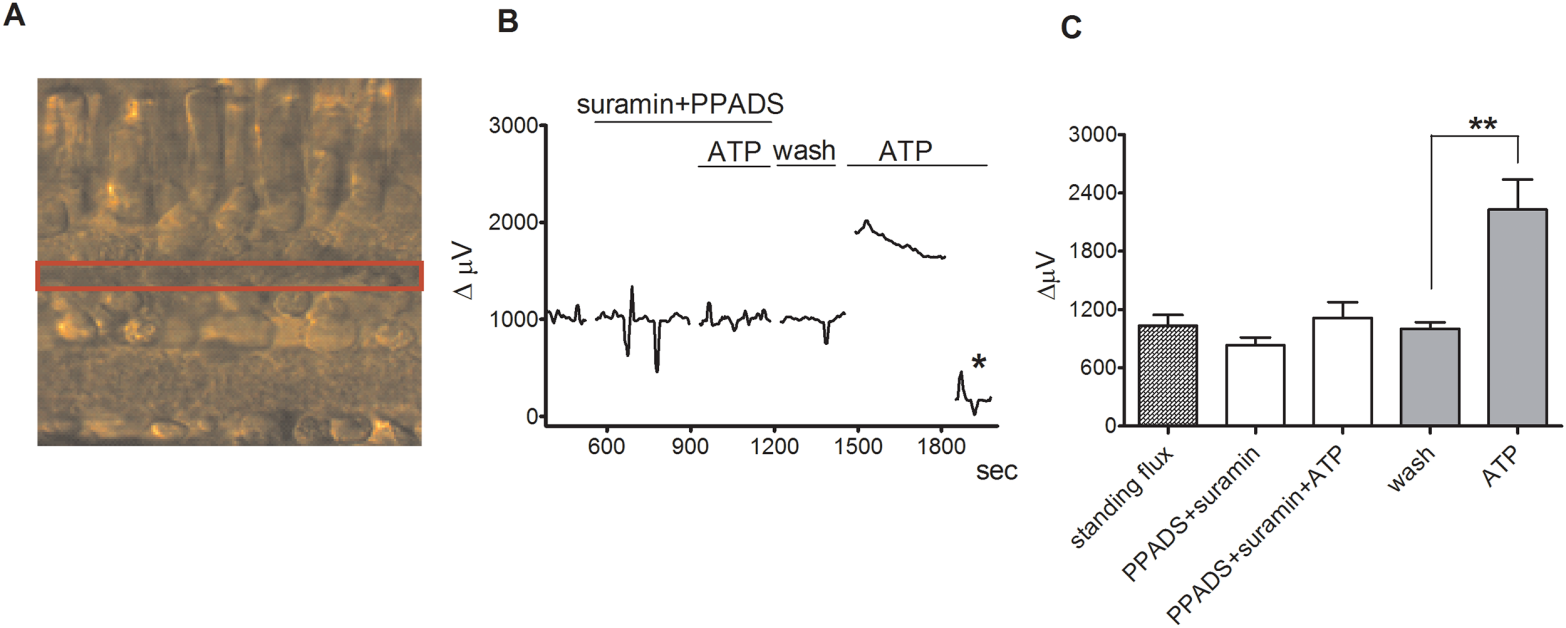
ATP-induces an increase in extracellular H+ flux at the outer plexiform layer (OPL) in retinal slices that is markedly reduced by PPADS and suramin. (A) Retinal cross-section of a tiger salamander retina; red box highlights the outer plexiform layer (OPL). (B) A representative trace showing the response observed to application of 100 μM ATP from an individual self-referencing recording from a retinal slice with the microelectrode positioned just above the OPL; asterisk indicates a background control reading taken 600 μm above the retinal slice. (C) Mean data from eight slices. ATP induced a significantly smaller increase in extracellular H+ flux in the background of suramin and PPADS than it did in plain Ringer’s solution.

Next, we examined changes in extracellular H+ fluxes from retinal slices in response to exogenously applied ATP (Fig 5). The H+-selective microelectrode was positioned several microns above the outer plexiform layer and a reference point was taken 30 μm away in a vertical direction. We examined responses to application of 100 μM ATP, with the expectation that ecto-ATPases present in the tissue would likely reduce the actual concentration reaching the tissue. In eight slices, 100 μM ATP caused a robust increase in extracellular H+ flux. The mean response increased from a standing, already acidic flux of 1000 ± 67 μV to 2227 ± 313 μV, p = 0.002. The response to ATP was significantly reduced in the presence of 300 μM of the ATP receptor blockers suramin and PPADS. Application of PPADS and suramin alone at 300 μM caused a slight decrease in the standing H+ flux from 1032 ± 111 μV to 832 ± 78 μV (p = 0.08). Addition of 100 μM ATP in the presence of pPADS and suramin produced a small increase in H+ flux (1112 ± 164 μV, p = 0.048). However, the response induced by ATP in the absence of PPADS and suramin was much larger than that induced when the blockers were present (p = 0.002). Similar results were obtained from goldfish slices, where H+-sensitive microelectrodes positioned above the outer plexiform layer detected a robust extracellular acidification induced by 100 μM ATP. The average H+ flux at the level of the outer plexiform layer prior to stimulation was 1205 ± 90 μV from six slices, and decreased to 861 ± 34 μV after the application of 200 μM suramin and PPADS (p = 0.003). Application of 100 μM ATP in the presence of PPADS and suramin caused a slight increase in the signal to 1064 ± 99 μV (p = 0.036). However, ATP caused a much higher H+ flux after PPADS and suramin were washed out; the signal increased from 968 ± 51 μV after wash-out of the drugs to 1856 ± 87 μV upon application of 100 μM ATP, p = 0.0001.

**Fig 6:**
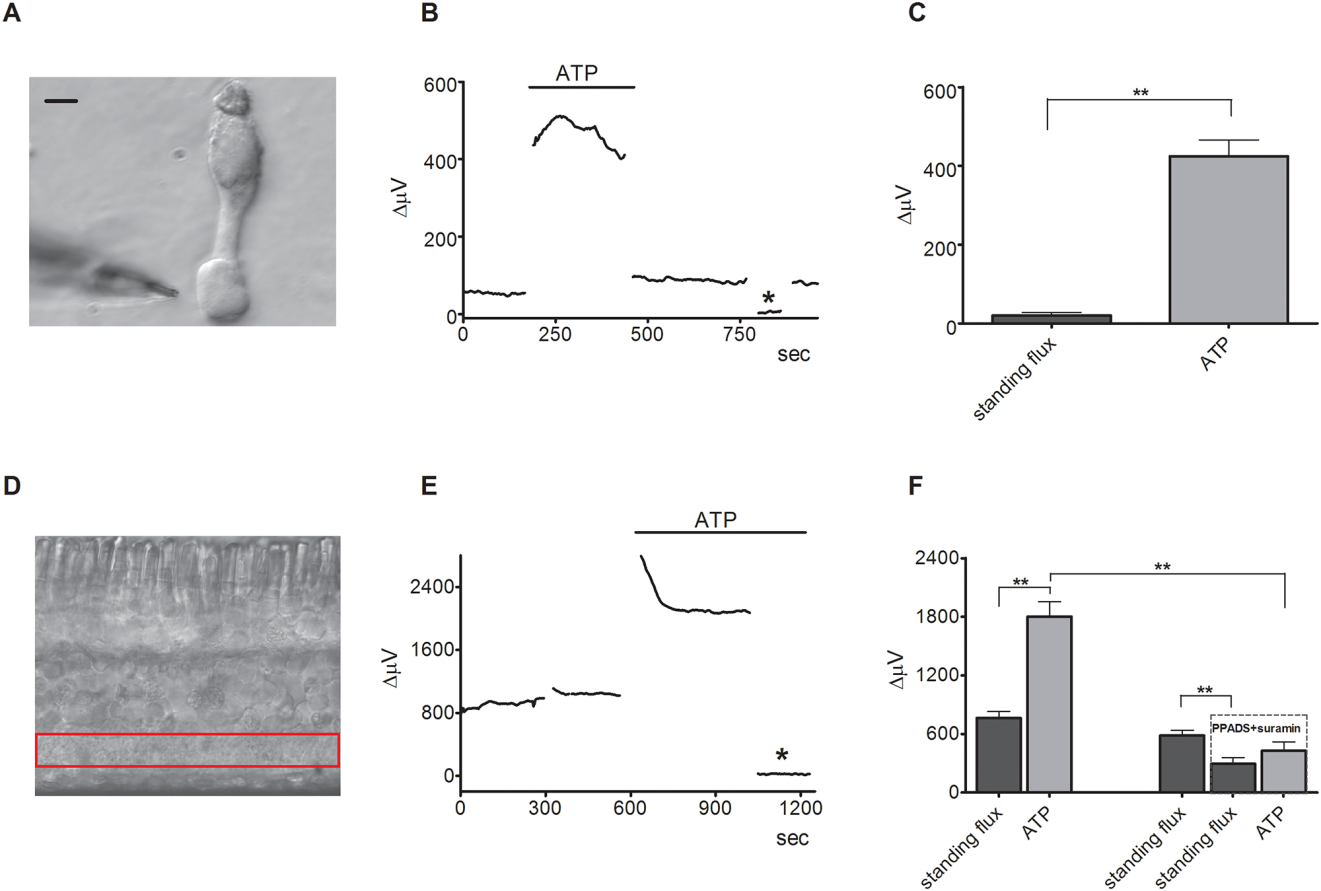
Extracellular ATP also induces increases in extracellular H+ fluxes from the end feet of isolated Müller cells and the inner plexiform layer in retinal slices that are reduced by PPADS and suramin. (A) An isolated Müller cell with an H+-selective microelectrode positioned by the end foot of the cell; scale bar, 20 μm. (B) A representative trace showing the increase in H+ flux measured from the end foot of a Müller cell in response to application of 100 μM ATP. (C) Mean responses of isolated Müller cells to 100 μM ATP; N = 9, asterisk denotes background control taken 200 μm above the cell. (D) Retinal slice with red box highlighting the inner plexiform layer (IPL). (E) A representative trace showing the response observed to application of 100 μM ATP from a self-referencing H+-selective microelectrode from a retinal slice when the microelectrode was positioned just above the IPL; asterisk indicates a background control reading taken 600 μm above the retinal slice. (F) Mean data from two populations; ATP induced a significant increase in H+ flux that was significantly smaller in the presence of suramin and PPADS (N = 6) than it did in normal Ringer’s solution (N = 6). The data from the two populations are separated by the gap on the x-axis and were compared via an unpaired 2-way t test.

We also found changes in H+ flux in response to bath-applied ATP at the end feet of acutely isolated Muller cells as well as at the level of the inner plexiform layer (IPL) in retinal slices (Fig 6). For recordings from isolated cells, the H+-selective microelectrode was positioned next to the end feet of Muller cells (Fig 6A). Bath application of 100 μM ATP caused a significant increase in extracellular H+ flux from the end feet of nine Muller cells (Fig 6B and 6C). On average, the H+ flux increased from 21 ± 7 μV before ATP was applied to 424 ± 42 μV after application of 100 μM ATP (p<0.0001). In slices, ATP also caused a robust increase in the extracellular H+ flux at the level of the inner plexiform layer (Fig 6D–6F) similar to the ATP-induced responses detected at the outer plexiform layer (Fig 5). The H+-selective microelectrode was positioned just above the inner plexiform layer (IPL) and moved to a reference location 30 μm vertically above the recording location. The mean H+ flux from six slices increased from 763 ± 68 μV to 1802 ± 151 μV after the application of 100 μM ATP (p = 0.0002). A control application of Ringer’s solution alone did not cause any significant change in H+ flux. Bath application of ATP to a second population of six slices in the presence of the P2Y receptor blockers PPADS and suramin, resulted in a much smaller increase in the extracellular H+ flux (Fig 6F). The application of 300 μM PPADS and suramin alone also significantly reduced the standing H+ flux when compared to the application of Ringer’s solution. The standing H+ flux decreased from 582 ± 55 μV in normal Ringer’s to 295 ± 65 μV in the presence of the two P2Y receptor blockers (p = 0.0002). Subsequent application of 100 μM ATP in the presence of the blockers increased the H+ flux only to 428 ± 91 μV (p = 0.012). The amplitude of the response to ATP in the presence of PPADS and suramin was significantly smaller than the ATP-induced change in H+ flux observed in the absence of the blockers (Fig 6F) (p<0.0001).

## Discussion

Our data demonstrate that extracellular ATP induces a pronounced and consistent increase in extracellular H+ flux from Müller cells of the tiger salamander and Müller cells of a number of other species, including human. The accumulated evidence suggests that the increase in extracellular H+ flux detected from activated tiger salamander Müller cells is mediated by activation of metabotropic P2 receptors leading to increases in intracellular calcium via an IP3-dependent pathway. We believe that these results have significant implications for the role that alterations in extracellular H+ play in retinal function.

Small alterations in the extracellular concentration of H+ have profound modulatory effects on neuronal transmission within the retina. Kleinschmidt [32] demonstrated that altering extracellular pH from 7.8 to 7.2 virtually abolished synaptic transmission from photoreceptors to second order cells in the retina of the axolotl, and Barnes and colleagues [9,33] provided convincing evidence that the modulation by extracellular H+ was mediated by a reduction in calcium entry through the L-type voltage-gated calcium channels on the synaptic terminals of photoreceptors in tiger salamander. DeVries [34] reported that the small number of protons emerging from synaptic vesicles undergoing exocytosis could inhibit neurotransmitter release from photoreceptors by blocking calcium influx through nearby calcium channels. The ATP-induced increase in H+ flux from Müller cells we report here has the potential to act as a key molecular mechanism mediating neuronal inhibition in the retina in general and in the outer plexiform layer specifically. Consistent with this hypothesis is the observation that extracellular ATP induced an increase in H+ flux at the level of the outer plexiform layer in intact retinal slices. The magnitude of the ATP-induced change in H+ flux detected from isolated Müller cells and in retinal slices with our self-referencing electrode system is on the same order of magnitude as that detected from cells and tissues known to be potent proton pumpers, such as in the vas deferens [35], where changes in extracellular H+ concentration play an essential role in activation of sperm. The ATP-induced increase in extracellular H+ flux we detect in the outer plexiform layer of retinal slices seems likely to be largely mediated by Müller cells; self-referencing recordings with H+-selective microelectrodes from photoreceptors isolated from tiger salamander have not shown any alteration in extracellular H+ flux upon application of 100 μM ATP, nor have we observed ATP-induced alterations in extracellular H+ flux from horizontal cells of catfish, or from horizontal cells, bipolar cells or photoreceptors of goldfish. The inability to easily identify and record H+ fluxes from isolated ganglion and amacrine cells leaves open the question of whether and how much these cells contribute to extracellular changes in H+ in the inner synaptic layer upon stimulation by extracellular ATP. Selective stimulation of Müller cells expressing DREADDs (designer receptors) combined with measurements of altered H+ flux would help to clarify the role that changes in extracellular H+ mediated specifically by Müller cells play in shaping retinal responses.

A recent study suggests that extracellular ATP exerts its effects on synaptic transmission in the retina by increasing pH buffering in the synaptic cleft [36]. The significant increase in extracellular H+ flux we have detected from isolated Müller cells and in retinal slices cannot be explained by such a mechanism. The reduction of the increase in extracellular H+ flux by the P2 antagonists suramin and PPADS strongly implicate that activation of ATP receptors are involved in the increase in H+ flux; the reduction by agents that block rises in intracellular calcium also do not fit the buffering hypothesis. Significant increases in extracellular H+ fluxes are also observed at concentrations of extracellular ATP as low as 1 μM, a concentration not likely to alter significantly the pH buffering capacity in our experimental conditions. The block of ATP-induced increases in extracellular H+ flux by the anion transport inhibitor DIDS also cannot be easily explained by simple increases in extracellular pH buffering. Finally, the extraceullar H+ flux is also elicited by the non-hydrolyzable ATP analogue ATPγs. Taken together, these results suggest that the observed changes in extracellular H+ flux from Müller cells cannot be accounted for by altered pH buffering by ATP.

Müller cells are known to possess a voltage-sensitive, sodium coupled bicarbonate transporter that has been shown to be capable of acidifying the extracellular fluid around isolated cells [30,37], and we have recently reported the characterization of this transporter using self-referencing H+-selective electrodes [19]. However, the bicarbonate transporter is unlikely to account for the acidification we detect, since the experiments reported here were all done in solutions lacking added bicarbonate and in which 1 mM HEPES was the only pH buffer present. Further, challenging Müller cells with 50 mM extracellular potassium (known to activate the voltage-dependent bicarbonate transporter and alter intracellular and extracellular levels of H+ in Müller cells [19,30] did not induce any significant alteration in proton flux from cells, again arguing against a contribution from this transporter. Finally, DIDS and SITS are both effective in blocking the acidifying effect of the sodium-coupled bicarbonate transporter [19], while the present study shows that the extracellular H+ fluxes reported here are blocked by 300 μM DIDS but not by a comparable concentration of SITS. While we do not yet know precisely what molecular mechanism(s) or transporter(s) are responsible for the observed extracellular H+ flux, the block by DIDS suggests a possible link to an anion-coupled transport system, although caution is needed even with this simple assessment, since DIDS has been suggested to be able to inhibit ATP-activated receptors directly [38]. It is unlikely, though, that the effect of DIDS is mediated by block of ATP receptors. ATP-induced intracellular calcium rises persist in the presence of DIDS, yet the ATP-induced increase in extracellular H+ flux is virtually abolished, suggesting that DIDS interferes with a step downstream from the activation of ATP receptors and ATP-induced intracellular calcium rises.

The high sensitivity of photoreceptor calcium channels to small changes in extracellular H+ has led to the hypothesis that the surround portion of the classic center-surround receptive fields of retinal neurons may be due to alterations in extracellular acidity mediated by retinal horizontal cells [6,39]. According to this hypothesis, glutamate released tonically by photoreceptors depolarizes horizontal cells; the depolarization leads to an increased release of protons from horizontal cells and inhibition of calcium influx through the photoreceptor calcium channels, with a consequent reduction in the release of glutamate. Two recent sets of experimental results have been taken as particularly strong evidence in support of the hypothesis that depolarization of horizontal cells induces an acidification in the synaptic cleft where photoreceptors contact second order cells. First, simultaneous paired recordings from horizontal and photoreceptor cells in retinal slices by Thoreson and colleagues reveal that direct depolarization of horizontal cells induces a rightward shift of the calcium conductance activation curve of photoreceptors that is abolished by enhancing the extracellular pH buffering capacity of the solution [40]. Additionally, Kramer and colleagues fused a pH-sensitive form of GFP (pHluorin) onto the extracellular side of a cone calcium channel subunit (dubbing the compound “calipHluorin”) and measured light-elicited changes in fluorescent intensity indicative of an acidification when horizontal cells are expected to be depolarized [11]. An extracellular acidification reported by calipHluorin in the photoreceptors was also observed in retinal slices when genetically modified zebrafish horizontal cells containing FMRF-amide receptors were depolarized by adding FMRF-amide. We hypothesize that these results may be accounted for by indirect activation of the neighboring Müller cells. Synaptic vesicles are known to possess high concentrations of ATP which can be released to the extracellular space by fusion of the vesicles with the plasma membrane (cf [41–43] for review). Although horizontal cells do not make significant numbers of conventional chemical synapses onto the terminals of photoreceptors in most species examined, they do make numerous conventional chemical synapses with bipolar cells [44,45]. Voltage-dependent extrusion of ATP could also result from release through voltage-sensitive pannexin channels present in the horizontal cells [36]. Depolarization of horizontal cells may thus be expected to lead to the extracellular release of ATP and indirect activation of neighboring Müller cell processes, with a consequent increase in Müller cell-mediated extracellular acidification. It remains an open question whether the changes in extracellular H+ mediated by Müller cells are of sufficient magnitude and rapidity to account for certain aspects typically equated with feedback inhibition onto photoreceptors (cf. [46]).

It is likely that horizontal cells are not the sole source of alterations in extracellular ATP in the vertebrate retina. Rather, vesicular release of neurotransmitters from virtually all neuronal subtypes is likely to be accompanied by concomitant release of extracellular ATP [41–43], with consequent alterations in extracellular H+ mediated by Müller cells. Our data showing ATP-induced modulation of H+ flux at the level of the inner plexiform layer supports this hypothesis. Such ATP-mediated changes in Müller cell H+ efflux have the potential to affect calcium-dependent neurotransmitter release from many different classes of neurons at many retinal levels. One interesting question is whether H+ efflux mediated by ATP activation of Müller cells would occur globally across the entire surface of a Müller cell, or whether instead local “hot spots” of H+ extrusion might take place. The growing body of evidence supporting the presence of microdomains of glial cell modulation [1,2] suggests the possibility that glial cell-mediated changes in extracellular H+ could be highly localized and might modulate highly restricted and independent sets of synaptic connections. Support for the notion of independent calcium domains within tiger salamander Müller cells includes our own previous experiments examining the effects of intracellular calcium rises induced by high extracellular potassium [19]. When tiger salamander Müller cells were tested in the same conditions as used in the present experiments (HEPES as the extracellular pH buffer with no bicarbonate present), high extracellular potassium levels induced significant increases in intracellular calcium that did not lead to any significant alteration in extracellular H+ flux. The potassium-induced increases in intracellular calcium required the presence of extracellular calcium. This is in striking contrast to our present observations showing that the ATP-induced increase in H+ flux required a rise in intracellular calcium that appears to arise from intracellular stores.

If the above line of reasoning is correct, what then is the likely physiological impact of protons released by the Müller cells? We suggest two important possibilities. First, Müller cells possess a glutamate transporter thought to remove glutamate from the synaptic cleft and keep extracellular glutamate levels low. This transporter when activated induces a rapid extracellular alkalinization likely due to cotransport of protons with glutamate into the cell (cf [47] for review). Removal of protons within the small space of the synaptic cleft has the potential to limit further the uptake of glutamate. It may be that Müller cells provide extracellular protons via the mechanism outlined in the present report in part to ensure continued removal of excess glutamate. Second, we hypothesize that H+ release from retinal Müller cells acts as an essential feedback mechanism to limit over-excitability in the retina. According to this idea, ATP co-released with glutamate from photoreceptor synaptic vesicles and potentially other synaptic terminals activates neighboring Müller cells, acidifying the extracellular solution; the protons would bind to photoreceptor calcium channels, reduce calcium influx, decrease vesicular fusion and depress the release of the excitatory neurotransmitter glutamate. We believe this mechanism can potentially explain an aspect of retinal function that has long puzzled retinal physiologists. Photoreceptors in the dark are believed to be tonically depolarized and constantly liberating glutamate; why should this release be highest in the dark, when we are asleep, and why does this expected continuous release of neurotransmitter not induce glutamate-mediated neuronal excitotoxicity, as observed in other areas of the brain when high levels of glutamate are present? We propose that glutamate release from tonically depolarized photoreceptors and other neurons is accompanied by co-release of ATP, leading to extracellular acidification mediated by Müller cells, and hypothesize that this release of acid from Müller cells acts as a form of automatic gain control, decreasing calcium influx into synaptic terminals and limiting neurotransmitter release from retinal neurons without altering the voltages of the cells themselves. Such control of synaptic gain by glial-mediated alterations in extracellular acidity is likely not restricted to the outer plexiform layer, but may occur in the inner plexiform layer as well, where bipolar cells send information to amacrine and ganglion cells: ATP-induced H+ flux from isolated Müller cells occurs not only at the apical tuft-cell body junction of Müller cells, but is detectable at other areas around the cell, and moreover, ATP elicits acidifications at the level of the inner plexiform layer as well in retinal slices.

There is considerable previous evidence that activation of Müller cells leads to modulation of retinal neurons [48–51]. The pattern of modulation is complex, with some classes of retinal neurons showing inhibition upon glial stimulation and others showing enhances in light-stimulated action potentials. In addition to uptake of neurotransmitter in the synaptic cleft by Müller cell transporters, multiple molecular mechanisms have been suggested to be involved in this modulation of neuronal activity by activation of Müller cells, with ATP playing a leading role but with evidence also for the involvement of glutamate, adenosine, GABA, glycine and serine. We believe our data strongly implicate ATP-mediated H+ release from Müller cells as a potentially important neuromodulator and gliotransmitter regulating synaptic transmission in the retina.

We further envision that H+-mediated gain control is not limited to Müller cells in the retina, but is likely to be a general mechanism used by glial cells throughout the brain to limit neuronal excitability. In support of this hypothesis, we have observed in preliminary self-referencing experiments that extracellular ATP also induces an increase in extracellular H+ from glial cells cultured from cortex and hippocampus. Our findings may impact on an additional question that has bedeviled the field of neuroscience for many years: what is/are the molecular mechanisms by which glial cells modulate excitability within the nervous system? We believe that our results point to H+ as a key “gliotransmitter” capable of modulating neuronal activity that may limit and prevent neuroexcitotoxicity within the nervous system.
